# Towards High-Throughput Chemobehavioural Phenomics in Neuropsychiatric Drug Discovery

**DOI:** 10.3390/md17060340

**Published:** 2019-06-06

**Authors:** Jason Henry, Donald Wlodkowic

**Affiliations:** The Phenomics Laboratory, School of Science, RMIT University, Melbourne, VIC 3000, Australia; s3740720@student.rmit.edu.au

**Keywords:** behaviour, phenomics, drug discovery, neuroactive, zebrafish, planarian

## Abstract

Identifying novel marine-derived neuroactive chemicals with therapeutic potential is difficult due to inherent complexities of the central nervous system (CNS), our limited understanding of the molecular foundations of neuro-psychiatric conditions, as well as the limited applications of effective high-throughput screening models that recapitulate functionalities of the intact CNS. Furthermore, nearly all neuro-modulating chemicals exhibit poorly characterized pleiotropic activities often referred to as polypharmacology. The latter renders conventional target-based in vitro screening approaches very difficult to accomplish. In this context, chemobehavioural phenotyping using innovative small organism models such as planarians and zebrafish represent powerful and highly integrative approaches to study the impact of new chemicals on central and peripheral nervous systems. In contrast to in vitro bioassays aimed predominantly at identification of chemicals acting on single targets, phenotypic chemobehavioural analysis allows for complex multi-target interactions to occur in combination with studies of polypharmacological effects of chemicals in a context of functional and intact milieu of the whole organism. In this review, we will outline recent advances in high-throughput chemobehavioural phenotyping and provide a future outlook on how those innovative methods can be utilized for rapidly screening and characterizing marine-derived compounds with prospective applications in neuropharmacology and psychosomatic medicine.

## 1. Introduction

**Figure 1:**
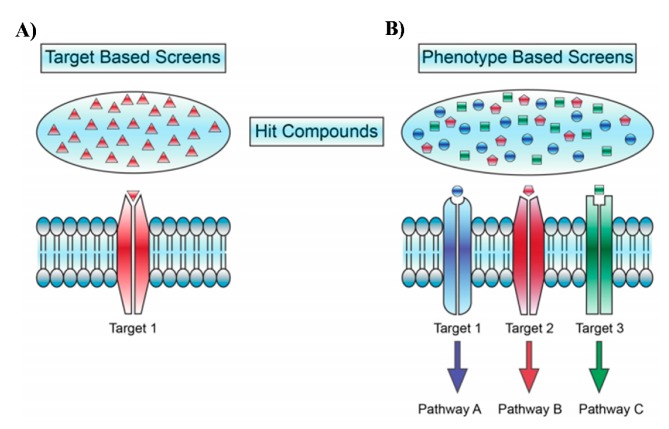
Phenotypic screening approaches in drug discovery embrace much larger molecular target space and provide an ability to study pleiotropic effects of drugs without *a priori* knowledge of molecular targets. (**A**) An example of molecular target-based assays. (**B**) An example of phenotype-based assays in drug discovery. Reprinted from [5] with permission from American Chemical Society, ACS Chem. Biol..

Contemporary drug discovery pipelines for most pathological conditions commonly employ molecular target-based screening of compound libraries. This approach is often referred to as reverse chemical biology/pharmacology and necessitates understanding of the mechanisms underlying the pathogenesis of a given disease with very well defined potential molecular targets for drug design and optimization (Figure 1) [1,2,3,4]. During last two decades the reverse pharmacology paradigm has become a mainstream approach in both the pharmaceutical industry and academic research superseding the previous forward chemical biology paradigm that employs phenotypic analysis (Figure 1) [1,2,3,4]. The reverse pharmacology is pragmatic and undoubtedly can provide very useful molecular target-based drug screening capability in many well-characterized diseases such as hyperplasia, hypertension or hyperlipidemia. Moreover the characterization of specific molecular targets helps in subsequent SAR optimization [4]. 

Unfortunately in recent years the above drug discovery paradigm has been plagued by some significant failures, especially in development of drugs for central nervous system (CNS) such as neurodegenerative and neuropsychiatric diseases [6,7,8,9]. Despite the urgent need for new and improved neuroprotective and neuro-psychiatric drugs, the discovery and clinical success rate of compounds targeting CNS is significantly lower than for any other therapeutic area [10,11,12]. Drug discovery of novel CNS-targeted therapeutics is particularly difficult due to inherent complexities of the central nervous system (CNS) interconnected functional aspects across many signalling pathways and organ systems [6,11,13]. Both our limited understanding of the molecular foundations of polygenic neuropsychiatric conditions and limited applications of effective high-throughput screening models that can recapitulate functionalities of the intact CNS profoundly hamper the discovery of CNS specific drugs [6,14]. Accordingly, in recent years there have been some spectacular failures of molecularly targeted drugs aimed at treatment of Parkinson’s and Alzheimer’s diseases [15]. Moreover, nearly all-existing neuro-psychiatric medicines in clinical practice today have been discovered in late 50s and 60s based on serendipitous observations of changes in behavioural phenotypes in humans and other animals [8,9,11,12]. To this date very little is actually known about actual molecular mechanisms underlying anxiety, depression, or schizophrenia while more complex psychosomatic conditions such as bipolar and borderline personality disorders are far from being understood clinically due to complex and heterogeneous manifestations [13,15,16,17,18,19]. 

Complicating the complexity of CNS drug discovery, the majority of neuro-modulating chemicals exhibit poorly characterized pleiotropic activities often referred to as polypharmacology [6,11,20]. The latter involves activation of many targets and renders molecular target-based in vitro screening approaches practically impossible to accomplish at our current stage of neurobiological knowledge [11,20,21,22].

It is, therefore, becoming apparent that complexity of CNS is likely to preclude mechanistic understanding of disease pathology in the foreseeable future [6,23]. The clinical practice in neuro-psychiatry demonstrates, however, that phenotypic drug discovery approaches despite all of their associated limitations can indeed be effective and are in fact the only current viable approach in the absence of complete understanding of disease pathology and underlying molecular mechanisms [6,13,16,23].

## 2. Phenotypic Screening in Neuro-Active Drug Discovery

An overarching objective of biomedicine is to understand comparative phenotypic manifestations of healthy versus diseased individuals [24]. A phenotypic manifestation occurs through a complex web of interactions between genotypes and environmental variables. The main goal of the pharmacological intervention in clinical practice is to correct pathological phenotypes [24]. This can be achieved without any precise understanding of the genetic or mechanistic foundations of the disease [1,3,4]. In contrast to reverse chemical biology, the phenotypic analysis identifies active compounds that induce a change in a cellular or physiological phenotype such as inhibiting cell death of dopaminergic neurons in Parkinson’s disease or normalizing a behavioural phenotype associated with a certain psychosomatic condition (Figure 1) [1,2,3,4,16]. Forward chemical biology paradigm in drug discovery, often referred to as classical pharmacology or phenotypic screening, does not rely on a priori knowledge or understanding of any molecular mechanisms (Figure 1) [1,2,3,4]. Protein targets can remain unknown even after the drug’s activity and efficacy have been determined [1,3,4]. 

During the last decade, an increasing number of authors have suggested that phenomics, broadly defined here as acquisition of organism-wide high-dimensional phenotypic data, will be a natural evolution and supplementation of existing molecular omics paradigms in drug discovery [6,24]. Phenomic-level data can help to understand genomic variants underlying phenotypes, pleiotropy of responses to pharmaceuticals as well as provide new high-throughput and content-rich screening paradigms in drug discovery [1,2,3,6,16,24].

Successful drug discovery from a phenotypic screening is not necessarily impeded by a lack of molecular insight. Phenotypic screening is usually more physiologically relevant and embraces the ability to study pleiotropic effects [1,2,3,6,16]. This can be, as clinical practice demonstrates, applied to the discovery of new drug especially against illnesses where our understanding of the underlying mechanisms is low, such as in the case of common psychosomatic conditions or neurological diseases [23]. All drug regulatory agencies readily approve any new drugs or drug combinations that are efficacious and safe for clinical use [1,2,4,25]. There are thus no specific regulatory requirements for elucidation of precise mechanisms of action or well-defined molecular targets [1,2,3,4]. Recent analysis of success rates in various clinical trials of small-molecule drugs has demonstrated that to date significantly more drugs were discovered and approved for clinical use using a phenotypic screening approach rather than molecular target-based approach [1,2,3,4,16].

## 3. Neurobehavioural Phenomics Using Small Model Organisms

There is a strong revival of interest in high-content and high-throughput phenomic approaches due to a significant number of setbacks, especially in neurobiology, in attempts to develop molecularly targeted therapeutics [6,16]. In order to rapidly screen thousands of chemicals for identification of their neuroactive potential, it is broadly understood that in vivo rodent models, although physiologically relevant, provide limited throughput and are associated with complex infrastructure, extreme costs, and ethical restrictions [16,26]. Accordingly, a large number of proxy phenotypic cell-based assays have been developed against an array of endpoints such as: neuronal differentiation, programmed cell death, neural progenitor cell (NPC) migration, neurite growth, neuron–glia interaction, synaptogenesis, and formation of neuronal networks and assessment of spontaneous neuronal activity using microelectrode arrays (MEAs) [27,28,29].

Unfortunately due to complexity of CNS the physiological relevance of cell-based assays is often difficult to quantify and translate to organism-level systemic neuropharmacology [6,13,16,30]. This is because even the most complex attempts at organotypic cell cultures do not represent truly integrated neural networks. As a result they are inherently unable to recapitulate the structural and functional integration of an intact CNS that manifests itself in complex endpoints such as behaviour and cognitive responses [6,16,23]. These responses are the ultimate result of neuronal networking and signaling and cannot be evaluated in vitro. Furthermore, nearly all neuro-modulating chemicals exhibit poorly characterized pleiotropic activities often referred to as polypharmacology [7,9,23]. This renders the conventional target-based in vitro screening approach very difficult to accomplish. Neuro-active therapeutics such as anxiolytics, antipsychotics, and antidepressants, alter the CNS function and thus manifestation of behavioural phenotypes and the latter can only be discovered using behavioural phenotyping [11,12,16,30,31].

**Figure 2:**
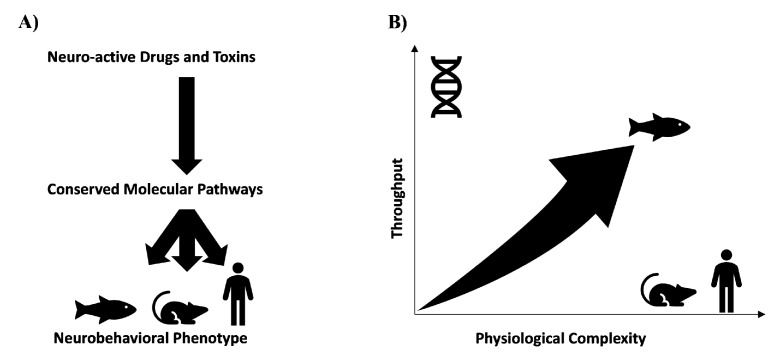
Chemobehavioural phenotypic screening utilizing small model organisms in neuropsychiatric drug discovery. (**A**) Utilization of small model organisms for high-throughput behavioural screening is possible thanks to evolutionary conservation of key neuronal pathways and molecular pathways. (**B**) Chemobehavioural phenotyping using innovative small organism models such as zebrafish (*Danio rerio*), fruit fly (*Drosophila melanogaster*), nematodes (*Caenorhabditis elegans*), and planarians (*Dugesia japonica* and *Schmidtea mediterranea*) can bridge the gap between conventional in vitro and in vivo drug screening paradigms. Small model organisms recapitulate the complexity of an intact CNS, while at the same time this can be performed in a higher throughput fashion and at a very low cost. Adapted from [9].

Since the behavioural phenotyping is an essential part of neuro-active drug discovery but cannot be efficiently performed on rodent in vivo models, there is a need for new models [31,32,33,34,35]. Such models need to recapitulate complexity of an intact CNS while at the same time can be performed in a higher throughput fashion [7,8,9]. In this context, chemobehavioural phenotyping using innovative small organism models such as zebrafish (*Danio rerio*), fruit fly (*Drosophila melanogaster*), nematodes (*Caenorhabditis elegans*), and planarians (*Dugesia japonica* and *Schmidtea mediterranea*) represent powerful and highly integrative approaches to study impact of new chemicals on central and peripheral nervous systems (Figure 2) [31,34,35,36,37,38,39,40]. Their advantages include relative low cost per screen, fast life cycles, ease of obtaining ethical approval, amenability to non-invasive, whole-animal in vivo imaging as well behavioural phenotypes that can be rapidly analysed [35,37,38,41]. Small model organisms offer intact, multicellular systems that model integrative biochemical and physiological processes, therefore providing an ideal model in a chemical screening system for drug discovery in neuropharmacology and neurotoxicology (Figure 2) [33,35,42,43]. In contrast to in vitro bioassays aimed predominantly at identification of chemicals acting on a handful of targets, phenotypic chemobehavioural analysis encompasses a much broader target space as well as enables studies of polypharmacological effects of chemicals in a context of functional milieu of the whole organism with an intact CNS [5,6,8]. Fundamental to use of small organism models is evolutionary conservation of neurodevelopment and neurophysiology [44]. In this regard many fundamental neurophysiology processes in these simpler model organisms are homologous to those in humans [35].

All small model organisms described above express orthologues of human genes known to be important in neuro-developmental disorders and have equivalent types of neurotransmitter signaling pathways to those in mammals [42,43]. These include: cholinergic, GABAergic, glutamatergic, dopaminergic, and serotonergic pathways [45,46]. Furthermore, since they share the common neurophysiological principles of locomotor network organization, they enable rapid screening of alterations in behavioural phenotypes [5,6,8]. In some cases analysis of cognitive functions in response to neuro-active chemicals is possible as demonstrated in zebrafish, fruit flies, and planarian models [38,47,48]. Apart from drug efficacy data, the phenotypic screens using in vivo model systems also include critical metabolic components and provide a physiologically relevant microenvironment that takes into account complex toxicokinetics of drugs that cannot be achieved in cell-based assays [34,35,41]. 

It is postulated that high-throughput chemobehavioural phenomics can become an important toolbox for discovery of novel neuro-psychiatric medicines, screening for neurotoxic side effects of drugs targeting CNS-unrelated pathways, specific nerve poisons and their antidotes, in addition to neuro-protective chemicals [5,32,49].

## 4. Examples of Chemobehavioural Models in CNS Drug Discovery

**Figure 3:**
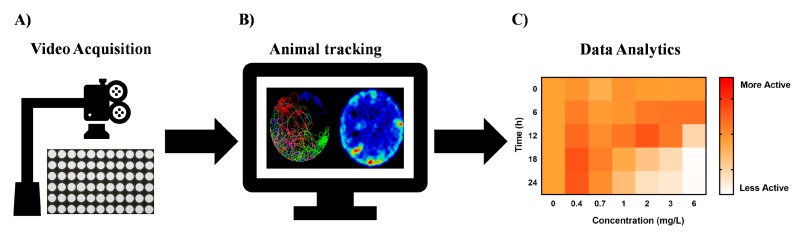
Key enablers for high-throughput chemobehavioural assays in neuroactive drug discovery. (**A**) Ultra-high-throughput video data recording systems to capture changes of behavioural phenotypes upon stimulation with chemical agents. (**B**) Automated animal tracking software capable of high-throughput data analysis from hundreds of samples. The organism’s behaviour is identified against the background of the vessel and run through quantifying tracking algorithms. (**C**) Systems-level phenotypic analytics to allow digitization of the behavioural traits into simpler biological fingerprints, phenotypic clustering, and elucidation of mechanism of actions.

Chemobehavioural phenotyping using innovative small organism models such as zebrafish, nematode (*C. elegans*), and planarians represent powerful and highly integrative approaches to study the impact of new chemicals on central and peripheral nervous systems [7,8,32,36] (Figure 2). Despite the advantages of small animal models, the development of new neuro-active chemicals is at present predominantly hampered by a lack of efficient high-throughput phenotype-based discovery technologies and methods [7,8,9,32,50] (Figure 3). To date, most pharmacological studies utilizing neurobehavioural screening techniques have been small in scale and limited in their objectives.

Below we outline a handful of notable examples of models utilized for screening of neuro-active chemicals. They provide opportunities for systematic discovery in the context of the intact nervous system through applications of neuro-behaviour phenotypes.

### 4.1. Behaviour-Based CNS Drug Discovery in Zebrafish

Zebrafish models have been widely used in a plethora of developmental biology and pharmacogenomics research. Zebrafish (Zf) genome exhibits over 71% similarity with human genes with its orthologs sharing up to 80% homology [42,51]. Importantly the zebrafish CNS shares significant structural homology with its mammalian counterparts in addition to very closely related molecular and physiological foundations of neuronal signaling [34,35,52,53]. Furthermore, the blood–brain barrier in zebrafish appears early in development and, similarly to that of higher vertebrates, is composed of endothelial cell tight junction [54]. 

From a standpoint of neuroactive drug discovery, embryonal, larval, and in particular adult stages of zebrafish can be effective in increasing our understanding of neuropharmacological modulation [5,7,8,9,49,51]. Adult stages exhibit higher-level complex neural and behavioural functions [44]. They therefore enable the establishment of many functional models to assess sensorimotor and cognitive functions. Adult Zf models have been demonstrated to exhibit complex psychosomatic deficits such as anxiety, depression, addiction, autism, and obsessive-compulsive states [44,55].

However, from a perspective of large screening capacity in early stages of drug discovery, only embryonic and larval stages can be realistically considered [56]. These early developmental stages can be kept in 96-well plates and low volumes of media thus providing high-throughput screening capabilities [34,35,52,53]. The behavioural functions can be then assessed using specialized video recording cameras imaging entire multi-well plates [57,58,59,60]. Animal tracking software suites are utilized to analyze and digitize behavioural phenotypes [57,58,59,60]. Visualization of neurodevelopmental processes utilizing a plethora of transgenic models is enabled by the transparency of embryo and larval stages. This highlights the possibility of creating multi-dimensional data that combine molecular and cellular assessments with the analysis of behavioural alterations [33,34,43,61].

**Figure 4:**
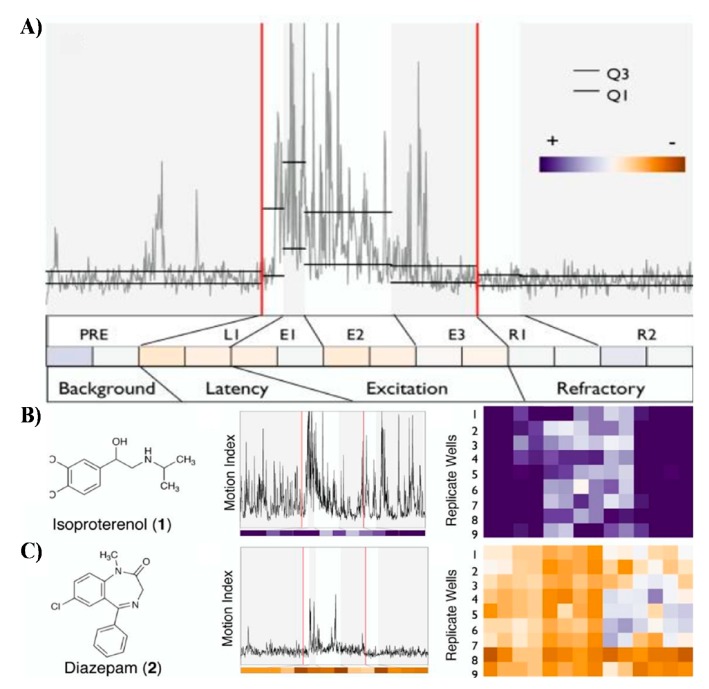
Photomotor response (PMR) assay performed on embryonic stages of zebrafish is an innovative high-throughput paradigm for discovery of new neuroactive chemicals. (**A**) Digital barcoding of stereotypic PMR responses from a representative untreated sample. The motion index profile was digitized into four phases spanning seven discrete periods: PRE, L1, E1, E2, E3, R1, R2. The 14-digit code formed the behavioural barcode used for the clustering of phenotypes. Colors in the heat map represent deviation from the average control phenotype: purple, higher activity; orange, lower activity. (**B**) Exposure to a psychostimulant, Isoproterenol, demonstrates increased embryo activity in PMR assay. (**C**) Exposure to an anxiolytic, Diazepam, demonstrates decreased embryo activity in PMR assay. Reprinted from [9] by permission of Springer Nature, Nature Chemical Biology.

Embryonic stages of zebrafish have been relatively unexplored models in discovery of neuroceuticals [5,7,8,9,49]. Recent discovery has shown that they exhibit stereotypic and reproducible photomotor responses (PMR) upon stimulation with high-intensity light stimulus (Figure 4) [9,32,45]. The PMR demonstrated by Kokel et al. can be used as a useful bioassay for high-throughput discovery of new neuroactive chemicals [9,45]. During the PMR assay embryos kept in darkness are exposed to chemicals in various exposure scenarios and before readout stimulated by a one second flash of high-intensity light (Figure 4). The latter induces the photomotor response characterized by a transient increase in frequency of spontaneous body flexions within their chorions lasting for about 5–7 s [9,45]. Kokel et al. demonstrated that PMR response can be translated into a relatively complex behavioural barcode that serves as phenotypic readout (Figure 4). Testing of a 14,000 natural-and-industrial small-molecule library validated that characteristic PMR barcodes can be used as signatures for categorization of diverse neuroactivities. For instance, psychostimulants increased PMR, whereas the majority of known anxiolytics decreased PMR readout. Finally, it appears that hierarchical clustering of PMR fingerprints with both known and uncharacterized chemicals can be used to discover new neuroactive drugs with similar modes of action to the existing ones [9,45].

**Figure 5:**
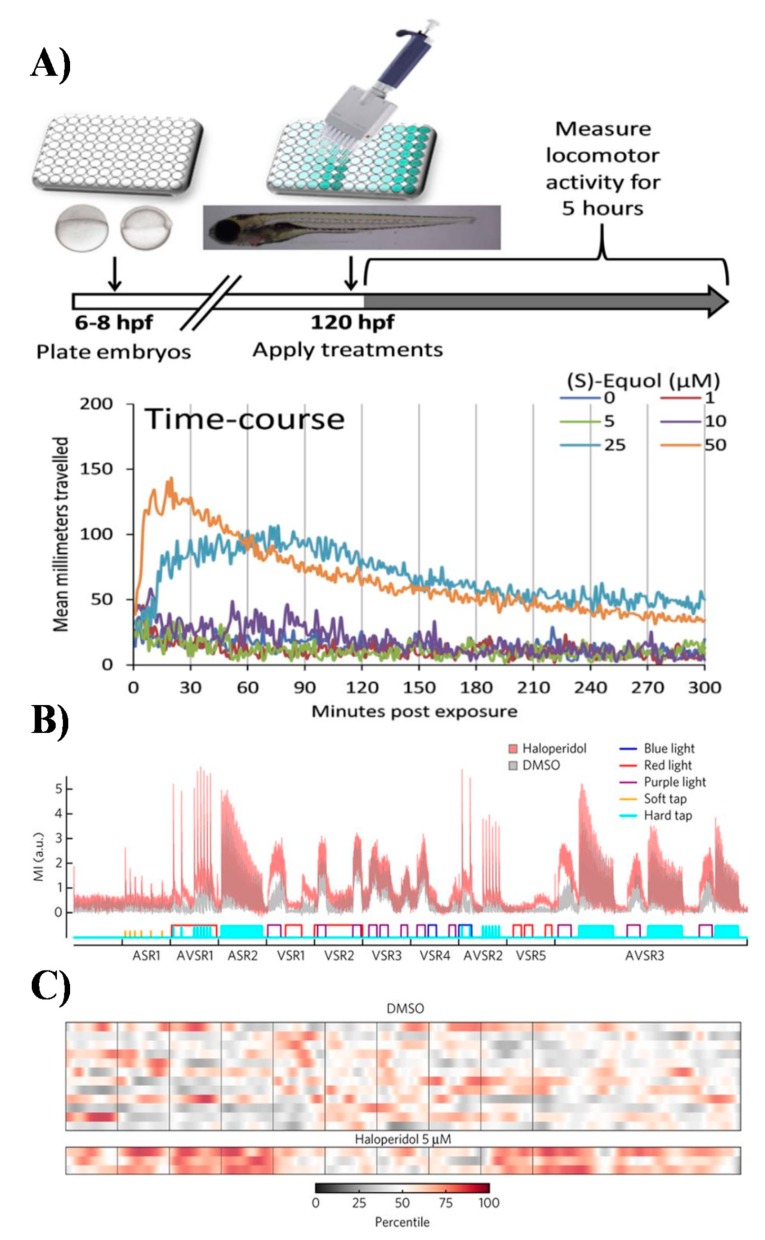
Stimuli-induced behaviours of zebrafish larvae in high-throughput neuroactive drug discovery. (**A**) Exposure paradigm and data analysis pipeline for the larval photomotor response (LPR) assay. Wild-type larval zebrafish were grown from 6 to 120 hpf, then exposed to test chemicals at 120 hpf to evaluate acute locomotor behavioural effects of neuropharmacologicals. Reprinted and adapted from [62] with permission from Elsevier. (**B**) A high-throughput larval zebrafish chemobehavioural test battery including two acoustic stimulus response assays, five visual stimulus response assays and three assays that combined acoustic and visual stimulus responses to systematically quantify effects of neuromodulatory compounds on vertebrate motor activity. The figure denotes digital fingerprints of larval zebrafish responses to control (DMSO) and Haloperidol. (**C**) Multidimensional behavioural assay on zebrafish larval stages elicited robust and reproducible patterns of behavioural phenotypes in larval zebrafish treated with neuroactive chemicals. For every drug treatment, each of the three rows represents a single test chamber containing 8 larvae. The x-axis indicates time and specific assays administered as shown in panel. Color-coding indicates percentile ranking of the motion index relative to DMSO controls. (**B)** and (**C)** reprinted from [8] by permission from Springer Nature, Nature Chemical Biology.

In contrast to embryos, the photomotor behaviour of zebrafish larvae (larval photomotory response assay; LPR) usually performed on stages between 4 to 5 days post-fertilisation (dpf) has been widely used to study neurotoxicity and pharmacological modulation of behaviours (Figure 5) [9,51]. One reason for this popularity is small size of the larval stages and ability to measure more advanced sensorimotor responses and non-associative learning repeatedly over time [62]. Larval stages exhibit stereotypic and reproducible startle responses to changes in cyclical light -> dark -> light stimuli (Figure 5) [62]. In particular, they display modest movements in the light phase, changing to a pronounced rapid increase (startle) in locomotion in the dark phase of the cyclic stimulus. The non-associative learning processes, such as habituation and sensitization, can be easily studied using LPR because zebrafish habituate to the photic stimulus over time [7,8,62].

The total distance traveled as well as swim speed and activity can be automatically derived from analysis of video files using commercial neurobehavioural analysis systems such as ZebraBox (ViewPoint Inc), and ZebraView (Noldus Inc). The rationale of the LPR assay is that any effects of neuropharmacological treatment will be visible in altered behavioural phenotypes that can be classified using known control drugs [7,8,62]. Importantly, automated behavioural phenotyping in larval stages can be also coupled with full-brain calcium imaging as well as performed in various transgenic lines and/or upon treatment with RNAi, creating great potential for improving resolution of behavioural profiles and elucidation of mode of action [63,64]. 

The LPR assay has been employed in many neuropharmacological studies and more recently was also for the first time used for characterization of marine natural products xyloketals and marine isoprenyl phenyl ether obtained from the mangrove fungus [7,8,65]. The latter was one of the very few studies that actually employed behavioural phenotyping using small model organisms in discovery of new marine-derived neuroceuticals [65].

Interestingly, despite common applications of LPR, very little development has actually been made in utilizing other sensorimotor assays such as combination of LPR, sensitivity to temperature (thermotaxis), vibration/touch (thigmotaxis), chemical gradients (chemotaxis), and phototaxis (Figure 5) [6,7,8]. It is anticipated that to generate rich behavioural profiles that can resolve complex and subtle differences between new neuroactive compounds, development of more advanced multidimensional phenotyping will be required [6,7,8]. In this regard, the recent work Bruni et al. has laid foundations for a battery of high-throughput behavioural assays to systematically quantify effects of neuromodulatory compounds on vertebrate motor activity [7,8]. The authors developed a test battery including two acoustic stimulus responses assays, five visual stimulus response assays and three assays that combined acoustic and visual stimulus responses. This multidimensional behavioural assay elicited robust and reproducible patterns of behavioural phenotypes in larval zebrafish treated with a set of known psychiatric drugs such as antipsychotics, antidepressants, and anxiolytics [7,8]. The reproducibility of behavioural phenotypes could be used to generate motion index (MI)-based fingerprints. The proof-of-concept of this approach was further demonstrated in discovery of antipsychotics and their mechanisms of action by screening a library of 24,760 compounds [7,8]. The library included 4,300 known bioactive compounds and 20,000 uncharacterized compounds with over 5,000 DMSO controls. The results were compiled in the first behavioural phenotypic database called PhenoBlast and used for behaviour-based connectivity mapping conceptually related to metrics used in existing fields of bioinformatics [7,8].

### 4.2. Behaviour-Based CNS Drug Discovery in C. elegans 

*C. elegans* has become a popular experimental animal, and even considering there is less genetic resemblance between nematodes and humans than other vertebrate models such as zebrafish, it has contributed greatly to the understanding of pathophysiology of human diseases [36,46,66,67]. *C. elegans* is very attractive because many inexpensive bioassays can be rapidly performed on small, rapidly developing worms that are amenable to high-throughput screening of drugs and toxins [36,67,68,69,70,71]. 

In the context of neurobiology *C. elegans* enables studies of neuronal development, connectivity, physiology, as well as alterations in behavioural traits, modulated by both genetic factors and pharmacological intervention [67,68,69,70,71,72]. Importantly, in contrast to zebrafish, it has a very well characterized nervous system with 302 neurons (divided into 118 distinct classes in addition to 56 glial cells), 1410 neuromuscular junctions, and 6393 chemical synapses (Avila et al., 2012) [67,71,73]. The synaptic transmission in *C. elegans* is also evolutionary, conserved with similar pathways to mammals including cholinergic, GABAergic, glutamatergic, dopaminergic, and serotonergic signaling [73,74]. This organism is so far the only one of which we possess an entire neuronal wiring chart mapping all neurons and major synaptic connections [73,74]. Despite the sheer simplicity of nematode’s CNS, it exhibits a variety of interesting behaviours such as: different motion and gait patterns, electrotaxis, chemotaxis, thermotaxis, light avoidance, mechanotransduction, learning, memory, mating, and sleep-like behaviours [66,67,72,75]. These can be used to elucidate the functions of neuronal pathways. Recent reports suggests that *C. elegans* is an exceptional model to explore molecular foundations of addiction and other drug-induced behavioural effects [66,67,71,72,76]. The optical transparency of worms also provide ample opportunities to combine behavioural data with real-time visualization of neurons in a plethora of transgenic lines and even study neuronal excitability using live-animal calcium imaging [77].

The simple structure of CNS combined with robust pathological-behavioural phenotypes has allowed *C. elegans* to become a valuable model for screening of drugs against age-associated neurodegeneration such as Alzheimer’s and Parkinson’s diseases [71]. Several recent studies demonstrated that phenotypic analysis of neurodegeneration can be modelled in worms and their applications can lead to accelerated drug discovery [78,79]. In relation to discovery of drugs for neuro-psychiatric conditions, *C. elegans* displays several conserved behavioural protophenotypes such as social feeding, immobility, startle suppression of pharyngeal pumping, and anorexic phenotypes [67,80,81]. Protophenotypes can be defined as precursors of higher CNS functions shared across species [82]. This conceptual framework of protophenotypes is currently fundamental to efforts aimed at deconvolution of psychiatric conditions into basic phenotypic clusters that could be then linked to underlying genetic and physiological configurations [67,83,84,85]. Accordingly, the evolutionary conserved protophenotypes can, in theory, be used in behaviour-based screening of psychosomatic drugs. While the worms do not experience any paranoid delusions or depression state they do show profound defects in food gathering motivation, social interactions, and overall apathy that are all fundamental symptoms translating to clinical manifestations in major depressive disorders (MDD) [67]. Certain loss-of-function genetic mutants have already been shown to exhibit schizophrenia-like deficits, while drugs such as antidepressants correct apathy (protophenotype of depression) in *C. elegans* [67]. 

In contrast to zebrafish, *C. elegans* has not yet been utilized for any large-scale screening routines in neuropsychiatric drug discovery. Nevertheless, there are some notable examples of small-scale behavioural screens that lay solid foundations for future research avenues. For instance, behaviour-based bioassays were recently used for discovery of selective suppressor of antipsychotic-induced hyperphagia and to identify drugs targeting tau neurotoxicity in Alzheimer’s disease and frontotemporal dementias [81,86]. In the latter study, using behavioural screens alone, McCormick et al. identified 16 candidates out of a library of 1120 that were effective in rescuing tauopathy-induced functional defects [86]. Importantly, this behaviour-based discovery in worms was further corroborated using knockdown studies in the human embryonic kidney/tau cell line thus providing validation of worm-based assays [86]. 

To enable broader adoption of behaviour-based drug discovery and high-throughput systematic phenotyping in *C. elegans*, large scale automated analysis methods require further refinement [41,70,87,88]. Standardized protocols for parameterizing worm behaviours are also slowly emerging. Only recently, Perni et al demonstrated a Field-of-View Nematode Tracking Platform (WF-NTP); an advanced machine vision system that enables massively parallel data acquisition and automated multi-parameter behavioural profiling on thousands of worms simultaneously [89]. Furthermore, Larch et al. and Nguyen et al. have demonstrated high-throughput in vivo imaging for simultaneous wide-field imaging of neuronal calcium activity in multiple freely moving worms [77,90]. Such bioanalytical systems allow for an unparalleled level of multiparameter phenotyping where real-time neuronal activity can be correlated with behavioural functions.

As demonstrated above, the development of novel high-throughput and multidimensional neurobehavioural screening in *C. elegans* provides a tantalizing glimpse on future drug discovery where digitisation of functional responses into fingerprints can be superimposed with cellular-level physiology for evaluating pharmacological modulation [88].

### 4.3. Behaviour-Based CNS Drug Discovery in Planarians

**Figure 6:**
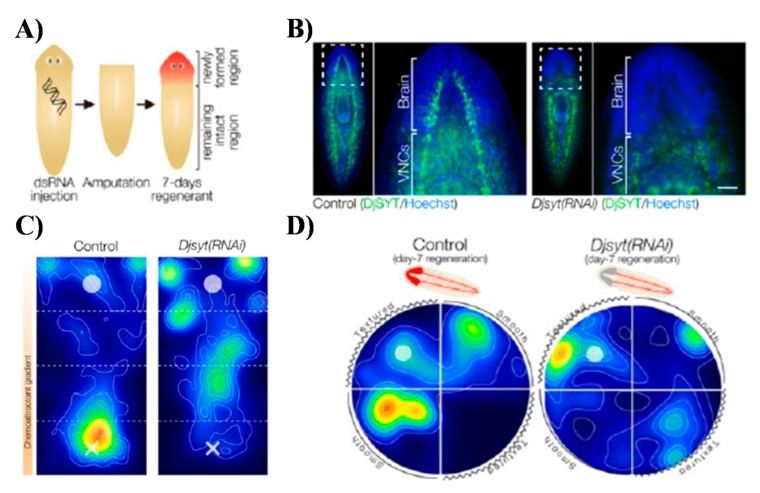
Planarians as neoclassical models in neuropharmacology and predictive neurotoxicology. (**A**) Regeneration of planarian CNS as well as straightforward applications of in vivo RNA interference in high throughput can be used to study of neuroregeneration pathways and pharmacological manipulations in brain injury. (**B**) Visualization of CNS regeneration using immunohistochemical detection of DjSYT protein (shown in green) in control and in Readyknock animals 7 days after decapitation. (**C**) Neurobehavioural analysis utilizing chemotactic behaviour of planarians in chemoattractant concentration gradient field. (**D**) Neurobehavioural analysis utilizing thigmotactic behaviour of planarians in structured environment. Reprinted and adapted from [104].

Planarians, often referred to as flatworms, are positioned between the *C. elegans* and zebrafish models in terms of evolutionary organismal complexity [38,47,91,92,93]. Similarly to previously described models, freshwater planarians are also small, very inexpensive to culture, breed, and develop usually within one week. They are best known from their phenomenal regeneration potential where even one adult planarian pluripotent stem cell (neoblast) can regenerate an entire functional organism [94,95,96]. This regenerative potential leads them to be fundamental models for regenerative medicine and healing research along with developmental and stem cell biology (Figure 6) [91,97].

From the perspective of neuroscience, planarians are the most primitive organisms that exhibit bilateral symmetry and cephalization with a fully centralized nervous system that features true synaptic transmission [93,98,99]. Their CNS is much more complex than that of nematodes, but simpler than that of zebrafish. Anatomically, it consists of a characteristic bi-lobed cephalic ganglion forming an inverted U-shape structure (commonly referred to as brain) and a pair of ventral nerve cords (VNC) along the longitudinal axis from head to tail (Figure 6) [93,98,99]. The CNS, despite its higher level of organizational and functional complexity, remains tractable on the cellular level with approximately 10000 identified neurons [93,98,99,100]. Interestingly, similar to vertebrates, the planarian brain acts as a central processing and decision-making unit. Accordingly, it features discrete anatomical regions responsible for distinct functionalities. Planarian CNS also features neuronal subpopulations and neurotransmitters such as acetylcholine, serotonin, glutamate, dopamine, GABA on top of a complex repertoire of over 100 distinct neuropeptides which are identical to those used by the mammalian brain [98,100]. Up to 95% of CNS-related genes in a popular planarian model organism *Dugesia japonica* have homologs in humans. Some reports have consequently postulated that planarians are unique among invertebrates in that they have a brain most similar to the vertebrates in terms of structure and function [101]. This results in planarians being a much more relevant model for uncovering key mechanisms of human brain function [101,102,103]. 

The relative complexity of the CNS combined with a complex sensory system ensures planarians exhibit a rich repertoire of stereotyped behavioural patterns [101,104]. These behaviours range with sensitivity of response to thermotaxis, chemotaxis, thigmotaxis, electric fields (electrotaxis), and magnetic fields (magnetotaxis) (Figure 6) [105,106,107]. They also exhibit learning and memory behaviours that can be altered following exposure to drugs affecting neural transmission [38,92,104]. For instance, when exposed to different addictive substances, including psychostimulant drugs, planarians display stereotyped mammalian-like behavioural responses including abstinence-induced withdrawal and anxiogenic-like response [108,109,110]. Both planarian phototactic as well as environmental place conditioning (EPC) behaviours were shown to be successful in studies of drug-seeking or anxiolytic-type phenotypes. The EPC bears a close resemblance to conditioned place preference (CPP) in rodents [103].

Planarian models could be effectively exploited in the neuropharmacology of drug addiction and in understanding the mechanisms of cognition and drugs that affect learning and memory when they are analyzed with high-throughput automated training and quantitative behavioural analysis systems [92,101,106,108,110,111,112]. They were in fact historically very popular models in neuropsychology and behavioural ethology, but interestingly, since around the 1960s, they were largely forgotten. At present there is a revival of interest in planarians as models in neurotoxicology and neuropharmacology [92,101,106,112]. It has been postulated that learning and memory paradigms demonstrated in planarians using automated and unbiased operant conditioning could be utilized to develop new behaviour-based screening paradigms for the development of the next generation of nootropic drugs (memory and enhancing chemicals) [38,92]. Considering their capacity to regenerate a fully functional brain upon amputation or injury, planarians can also become uniquely suited models for development of adult stem cell therapies that allow restoration of cognitive structures after injury (Figure 6) [91,102,113].

Most of the above still requires significant advancements in appropriate technologies in combination with the re-introduction of planarian models; however, this does offer some tantalizing prospects to applied neuropharmacology research and neuroactive drug discovery. No high-throughput or even low-throughput behavioural screens have been performed so far using these model organisms. Considering potential experimental advantages of those neoclassical models, however, we anticipate significant opportunities for neuroactive drug discovery procedures to be developed [38,47,91,92].

## 5. Key Enablers for High-Throughput Chemobehavioural Phenomics

As demonstrated above, high-throughput chemobehavioural phenotypic screens utilizing small model organisms can provide distinctive advantages in translational neuropharmacology [5,6,33,49]. This holistic approach with large target space enables discrete signatures, such as behavioural barcodes, to be used in identification of novel neuroactive chemicals. However, to fully leverage the potential of behaviour-modifying phenotypic screening paradigms and enable their broader adoption, several key aspects require further development and validation. Biological models, data acquisition, system-level analytics, and elucidation of the mode of action necessitate enhancing (Figure 3).

### 5.1. Biological Models

Refinement, standardization, and proper validation of biological models are fundamentally important in drug discovery. At present, analysis of neuro-behavioural alterations in response to pharmacological stimuli is conducted using non-standardized approaches, hence, comparative analysis and data mining between experiments performed in different conditions is difficult and prone to methodological errors. Furthermore, although small model organisms share a large number of neurophysiological characteristics with their mammalian counterparts, some significant inter-species differences in molecular, cellular, and functional characteristics exist. These models can lead to false positive or false negative hits due to molecular target differences (e.g., abundant vs. low expression of certain targets), species specificity with regards to exposure duration (e.g., some neuroactive chemicals will require longer receptor occupation in certain animals), as well as properties related to absorption, distribution, metabolism, excretion, and toxicity (ADMET; e.g., metabolism of some drugs is different in zebrafish than in humans) [8,9]. Since exposure of a small model organism in most high-throughput assays is performed using an immersion-based treatment, translation of experimental doses to clinical ones is often difficult [5,7,8,9]. For instance, in larval stages of zebrafish, many compounds frequently exhibit pharmacological effects in the μM range where high concentrations (>100 μM) are often toxic whereas low concentration (<100 nM) have no effects [5,7,8,9]. Furthermore it still remains unclear whether for instance assays performed on larval stages of zebrafish can provide sufficient phenotypic resolution to study complex and multitarget mechanisms of drugs, such as antipsychotics [5,6,7,8,9]. Proper development of biological models and validation against existing benchmarks can alleviate some of the above limitations and is fundamentally important for the future advancement of high-throughput neuro-behavioural screening routines.

### 5.2. Data Acquisition 

Cost and time saving plays an ever-increasing role in pharmaceutical screening routines. Initial efforts in behavioural phenomics should be put on development of standardized and user-friendly infrastructure. This is a fundamental enabler to the future of this budding field (Figure 3). Currently emphasis has been focused on automation of handling and analysis of different organisms, such as embryonic and larval stages of zebrafish and a plethora of small invertebrates [41,114,115,116]. However, progress in automated analysis of behaviours is significantly lagging behind [51,55,117,118]. This is because acquisition of data on behavioural traits requires sophisticated video recording systems coupled with specific stimulation modules (e.g., photic, chemical, thermal stimuli) [38,57,58,59,119]. Access to such specialized equipment is at present profoundly limited. As of 2019 only four manufacturers (ViewPoint Inc, Noldus Inc, Phylumtech Inc, Zantiks Inc) provide off-the-shelf equipment for basic neuro-behavioural analysis, and of these, two are just budding start-ups (Phylumtech Inc, Zantiks Inc). Unfortunately, most of the currently available equipment is not designed for applications in the high-throughput behavioural analysis nor is designed for studies of higher-level neurological functions such as associative learning (e.g., operant and classic conditioning) or 3D tracking of small rapidly moving animals [38,51]. As such many laboratories interested in this field of research must resort to cumbersome adaptations of existing automated microscopy and/or attempt to design and construct their own custom video recording systems [38,88,120]. 

In our view, the advancement of neurobehavioural phenomics depends heavily on development of high throughput imaging technologies that can maximize the number of samples and therefore substantially decrease the cost of screening routines (Figure 3) [6,24,88]. Currently, technology development in neurobehavioural research is stagnant and narrowly focused on the very basic needs of low-throughput experimentation with limited scope for modularity and innovative applications [37,38]. We suggest that development of new systems should focus on modular solutions that can readily be modified for use across different model species e.g., zebrafish, fruit fly, planarians. A particular care should be also given to design user-friendly interfaces so that scientists can focus on new discoveries and the refinement of the use of the biological models discussed above rather than trying to learn complex analytical apparatuses [37,38].

### 5.3. Systems-Level Analytics 

Another critical key enabling area for behavioural phenomics will be high-throughput and automated data analysis and data mining (Figure 3 and Figure 4) [121]. At present, analytical and bioinformatics approaches that can perform rapid analysis and mining of multi-dimensional behavioural data do not exist [32,49]. Automated techniques that go well beyond simple analysis of video files to reconstruct animal trajectories and basic behavioural parameters, such as speed of locomotion or distance travelled, will be necessary to elucidate patterns of behavioural responses, generate new behavioural barcodes, build phenoclusters, and even accelerate nonlinear system-level modeling (Figure 3) [6,32,49]. However, at present, even automated software techniques that can optimize and analyze hundreds of video files by using object tracking and movement trajectory reconstruction in a user-friendly and high-throughput manner are largely missing [121].

### 5.4. Elucidation of The Mode of Action 

A primary focus of phenotypic screening is identification of bioactive chemicals that change the pathological or chemically induced phenotype [49]. However, despite identification of a new behaviour-modifying compound, the question will still remain about the actual mode of action and molecular target identification. In this regard, it has been recently demonstrated that hierarchical clustering of behavioural fingerprints can indeed be used to identify neuroactive chemicals in addition to classifying them into functional classes independent of their known structure [6,9,45,49]. This reveals that systems-level phenotypic analytics of behaviours can be used to discover the mechanism of actions. As an example, using only phenoclusters of behavioural fingerprints, Kokel et al. could predict and then experimentally validate two new acetylcholinesterase (AChE) inhibitors, as well as new coumarin-like compounds with chemical similarity and activity to known monoamine oxidase inhibitors. Important to note is that some of the identified chemicals showed activity only in in vivo assays demonstrating significant advantages of in vivo model systems in neuroactive drug discovery [6,9,45].

As demonstrated above, using high throughput and multi-dimensional in vivo phenotyping should enable quite robust identification of compounds with enough statistical power to even derive target prediction [6,9,45]. We anticipate that development of system-level multi-dimensional analytical tools in behavioural phenomics combined with validated bioassay will be paramount to discovery of a plethora of new neuroactive chemicals [24,32].

## 6. Limitations of Behaviour-Based Drug Discovery

As briefly discussed above, there are many reasons why neuroactive drug discovery in small model organisms such as zebrafish may not directly translate into clinical practice. Issues such as target orthology, differences in blood−brain barrier, evolutionary divergence in certain neural pathways, ADMET, and differences in methods of drug delivery between small model organism tests and clinical administration can impede actual therapeutic predictions [33,56,122]. Our understanding of higher neural functions such as learning and cognition even in humans is very basic. A significant bottleneck arises, in particular during development of representative experimental proxies, to study the impact of neuro-modulating chemicals on associate learning and memory recall. However, such experiments can only be performed on model organisms with intact CNS, and as such, improvements in current methodologies are of paramount importance [33,56,122]. 

Commonly postulated as limitations of behaviour-based drug discovery are the significantly lower throughputs than those achieved in in vitro assays, higher overall costs, and difficulties in determining precise molecular mechanisms of action. This is often combined with the fact that behaviour is a complex and often multi-dimensional readout. In particular, the temporal and context dependent nature of behaviour combined with high variance present a significant challenge for design of robust experimental approaches [56]. The latter requires state-of-the-art high-throughput data recording and automated video analysis technologies that still remain underdeveloped [57,58,59,119]. Furthermore, because behaviours are complex and thus difficult to quantify, lack of established systems-level behavioural phenomics tools hampers digitization of the behavioural traits into simpler biological fingerprints [5,7,8,9,56]. 

Undoubtedly, all of the above points are valid and need improvements in development of new experimental approaches. Nevertheless, the existing collective body of evidence garnered on recent empirical results presents a compelling argument for, rather than against, further developments of behaviour-based drug discovery platforms in neuro-psychiatry [6,49].

## 7. Future Perspective

Despite a tremendous progress in development of small molecule and biological drugs over last two decades, the discovery and clinical success rate of CNS-targeted therapeutics is significantly lower than for other therapeutic areas. We have to appreciate that inherent complexity of the central nervous system and our still rather rudimentary understanding of higher brain functions will preclude mechanistic understanding of CNS pathologies in the foreseeable future. The clinical practice demonstrates, however, that behaviour-based drug discovery is not only possible but currently perhaps one of the very few viable approaches available in the absence of complete understanding of disease pathology.

Despite all claimed limitations often raised against phenotypic screening, we acknowledge that every biological model is, as the name suggests, only a proxy system. Every existing model whether cell-based or in vivo is thus burdened by a significant number of limitations and approximations that are indeed unavoidable. Existing in vitro biochemical and cell-based assays are inherently unable to recapitulate the structural and functional integration of intact CNS while at the same time mammalian models are too complex and costly to be implemented at a high throughput. Small model organisms will therefore become increasingly used as models of choice in neuroactive drug discovery. Compared to cell-based assays, systematic neurobehavioural phenotyping in small model living organisms can provide a more holistic understanding of functional neuropharmacology. This includes effects modulated by compound metabolism or polypharmacology induced by complex interactions with multiple biological pathways. In addition, considering recent empirical evidence, small model organisms appear to provide an optimal toolbox for behaviour-based discovery of novel chemicals that affect CNS functions. Existing limitations will require further research and development of new bioassays, standardization of test protocols, development of high-throughput behavioural data acquisition techniques, and even new systems-level analytics of content-rich phenotypic data. 

Ultimately, any compounds identified in small model organism screens will require further testing and refinement in rodent and other mammalian models to explore relevant pharmacokinetic properties, brain penetration, and behavioural effects. 

Nevertheless, behavioural phenomics can provide information rich data that recapitulates pleiotropic effects of drugs under relevant physiological conditions. When applied to CNS drug discovery, such datasets can potentially generate highly predictive in silico models based on fundamental biological discoveries. The value of neuro-behavioural phenomics lies in innovative and high-throughput approaches that can accelerate drug discovery for the conditions that suffered from very little therapeutic progress. Such avenues, despite all perceived limitations, simply cannot be ignored.
